# Development and validation of the Epilepsy Self‐Stigma Scale

**DOI:** 10.1002/epi4.12547

**Published:** 2021-10-26

**Authors:** Izumi Kuramochi, Takayuki Iwayama, Naoshi Horikawa, Sakie Shimotsu, Satsuki Watanabe, Hideo Yamanouchi, Haruo Yoshimasu

**Affiliations:** ^1^ Department of Psychiatry Saitama Medical Center Saitama Medical University Saitama Japan; ^2^ Saitama Medical University Comprehensive Epilepsy Center Saitama Japan; ^3^ Department of Psychology Showa Women's University Tokyo Japan; ^4^ Faculty of Human Development and Education Kyoto Women's University Kyoto Japan

**Keywords:** epilepsy, patients with epilepsy, scale, seizure, self‐stigma

## Abstract

**Objective:**

Self‐stigma is the internalization of negative public attitudes and is often experienced by patients with epilepsy (PWE). Greater self‐stigma is associated with lower self‐esteem and hinders therapeutic behavior. The study aims were to develop the Epilepsy Self‐Stigma Scale (ESSS) to assess self‐stigma in PWE and to examine the scale's reliability and validity.

**Methods:**

We created a test scale based on items from an existing stigma scale and the results of a previous qualitative analysis we conducted. We recruited 200 outpatients from departments specializing in epilepsy (psychiatry, neurology, and pediatric neurology) at four facilities in Tokyo and Saitama prefecture, Japan, between September and December 2020. Participants also completed the Rosenberg Self‐Esteem Scale (RSES) and Beck Depression Inventory (BDI‐II).

**Results:**

Questionnaires were returned from 102 participants (response rate: 51%). After excluding two participants with incomplete questionnaires, data for 100 participants were analyzed (53 women, 47 men; mean age [standard deviation]: 39.86 [17.45] years). Exploratory factor analysis extracted eight items loading on three factors: internalization of stigma, societal incomprehension, and confidentiality. Cronbach's α for all items and each factor demonstrated acceptable internal consistency (α = 0.76‐0.87). Test‐retest reliability was confirmed using data from 21 participants who completed the scale twice (r = 0.72 to 0.90). ESSS total scores and subscale scores correlated with RSES and BDI‐II scores (r = −0.30 to 0.55). The ESSS demonstrated substantial constructive validity. However, total scores did not significantly correlate with objective physician assessment of self‐stigma.

**Significance:**

The results showed that the eight‐item ESSS has high reliability and validity. This scale could facilitate the examination of factors associated with self‐stigma in PWE, which could inform the development of effective interventions for reducing stigma.

**Key Points**

- We created a questionnaire to measure self‐stigma in patients with epilepsy.
- We identified three self‐stigma factors: internalization of stigma, societal incomprehension, and confidentiality.
- Self‐stigma in patients with epilepsy did not correlate with objective physician evaluations.

## 1 INTRODUCTION

### 1.1 Epilepsy in Japan

The prevalence of patients with epilepsy (PWE) in Japan is approximately 0.8%, and the estimated number of patients is nearly 1 million.^1^ Epilepsy thus represents a substantial public health concern. Epileptic seizures are associated with unpredictable symptoms and can cause anxiety and fear in PWE and other people. Thus, epilepsy has historically engendered negative attitudes in many different cultures and has been associated with concepts such as madness and demons. For instance, the Japanese term for epilepsy “tenkan” has the negative meanings of “to become mad” and “a violent temperament that is apt to be infatuated.” Thus, the name itself is discriminatory. Although epilepsy is now understood to be caused by abnormal neuronal activity in the brain, discrimination and stigma against PWE remain deeply rooted in Japanese culture.^2^ Individuals with epilepsy may experience many types of prejudice, which may mean they are unable to find employment, are discouraged to participate in school events, and are not expected or permitted to marry. Understandably, such stigma can substantially affect the psychology and behavior of PWE.

### 1.2 Self‐stigma in PWE

Stigma associated with epilepsy is common in many cultures^3^ and considered one of the most important negative influences on the lives of PWE and their families.^4^, ^5^, ^6^, ^7^ Thornicroft defined stigma as a comprehensive concept that includes cognition, emotion, and behavior.^8^ Stigma can be broadly divided into public stigma, which is a societal prejudice against a disease, and self‐stigma, a prejudice that patients have against themselves.^9^ Individuals with schizophrenia and depression who have high self‐stigma regarding their illness are more likely to delay clinic or hospital visits, have difficulty continuing treatment, and have lower self‐esteem and self‐efficacy, all of which can hinder recovery from mental illness.^10^ Self‐stigma is a major factor in the treatment of PWE and can influence treatment effects, patient prognosis, and quality of life (QOL).

### 1.3 Development of the Epilepsy Self‐Stigma Scale (ESSS)

Questionnaire studies on felt stigma (feelings of stigma) and self‐stigma (internalized stigma) in PWE have recently been conducted worldwide.^11^, ^12^, ^13^, ^14^, ^15^, ^16^ Earlier reports on epilepsy‐related stigma, particularly in Europe, show that epilepsy‐induced social and occupational concerns, epilepsy status, and poor physical health tend to exacerbate patients’ perceived stigma. Cross‐cultural variation has also been reported.^11^, ^12^, ^13^ Frequent, severe seizures result in low self‐esteem, and awareness of employment discrimination and restrictions owing to disability and limited education are involved in the formation of stigma.^14^, ^15^ A survey of children with epilepsy found that negative attitudes, anxiety, low self‐esteem, and depression were associated with perceived stigma.^16^ Although epileptic seizures are acute clinical symptoms, PWE often experience internalized stigma following a seizure.^17^ Greater perceived courtesy stigma (ie stigma from being related to a person with stigma) in family members of PWE is associated with patient self‐stigma and QOL.^18^ Previous studies also suggest a strong link between patient self‐stigma and QOL; however, methods for reducing self‐stigma and increasing coping remain unclear and require a more comprehensive understanding.

An eight‐item version of the Stigma Scale for Chronic Illness (SSCI‐8) has been developed to assess the internalized stigma of chronic diseases.^19^ PWE are more likely to internalize stigma than individuals with other chronic neurological disorders (eg multiple sclerosis, Parkinson's disease, and amyotrophic lateral sclerosis).^19^ In previous studies, there were various self‐perceived stigma in epilepsy scales have been created in each country.^20^, ^21^, ^22^ However, there is no questionnaire to objectively judge the strength and content of their own self‐stigma (not social stigma, focusing on self‐stigma only) created by extracting the patient's own self‐stigma. The present study aim was to develop and validate a tool to measure perceived self‐stigma among PWE.

## 2 METHODS

We developed the scale items in two steps. First, we created a test scale of 18 items based on the results of a qualitative analysis we conducted,^2^ referring to the wording and structure of existing scales as necessary. Second, we administered the test scale to PWE to examine its reliability and validity.

### 2.1 Generation of scale items

We developed prototype scale items to measure the extent of self‐stigma in PWE, using categories derived from the results of our previous qualitative study.^2^ The purpose of this study was to investigate the quality and degree of cognition regarding self‐stigma in PWE. We conducted semistructured interviews with 20 epilepsy patients aged 20‐65 years who visited our psychiatric outpatient clinic. Using the interview data, the self‐stigma experiences of PWE were classified into 22 minor categories (eg “Negative cognition regarding epileptic seizures,” “It is difficult to tell others that I have epilepsy,” and “Social prejudice related to epilepsy as a disease and the diagnosis of epilepsy”), and 3 major categories (“self‐stigma,” “cognition regarding public stigma,” “anxiety and distress”). Using these categories, an 18‐item test scale was developed. The scale items were created to include the concept of the codes, consisting of many verbatim data. When necessary, we reviewed previous studies and referred to existing scale items.^14^, ^15^, ^16^, ^17^, ^20^, ^21^, ^22^, ^23^ To confirm the scale's content validity, we consulted two epileptologists, a clinician experienced in creating psychometric scales and two clinical psychologists working in the mental health field. Both epileptologists are qualified as psychiatrists, and the clinical psychologists are qualified as certified psychologists in Japan. Following their suggestions, we modified the expression of some items. Using this process, an 18‐item prototype scale was created.

### 2.2 Participants and procedure

We recruited 200 outpatients from departments specializing in epilepsy (psychiatry, neurology, and pediatric neurology) at four facilities in Tokyo and Saitama prefectures, Japan. The questionnaire was distributed during clinic visits and returned by mail. The survey was conducted between September and December 2020.

### 2.3 Measures

#### 2.3.1 Demographics

The following patient information was collected: age, sex, educational history, employment, marital status, and living style.

#### 2.3.2 Epilepsy Self‐Stigma Scale (ESSS)

The prototype scale comprised 18 items rated on a four‐point Likert‐type scale: 1: strongly disagree, 2: disagree, 3: agree, and 4: strongly agree. Total scores ranged from 18 to 72. Higher scores indicate greater self‐stigma caused by epilepsy. Although the original draft scale was in Japanese, an English version was developed using reverse translation by multiple experts. A full list of items in the prototype version is shown in Table S1.

#### 2.3.3 Rosenberg Self‐Esteem Scale (RSES)

Self‐stigma is negatively correlated with self‐esteem.^10^, ^17^ In the present study, self‐esteem was measured using the RSES^24^, ^25^ to examine the construct validity of the ESSS. Items are rated on a five‐point Likert scale from 1 (strongly disagree) to 5 (strongly agree). Total scores range from 10 to 50. Higher scores indicate higher levels of self‐esteem. We used the Japanese version of the RSES,^26^ which has been confirmed as having high validity and reliability using back translation. (Cronbach's alpha for the original Japanese version of the scale is 0.81.) In the present study, the RSES showed high internal consistency (Cronbach's α = 0.89).

#### 2.3.4 Beck Depression Inventory‐Second Edition (BDI‐II)

Self‐stigma is positively correlated with depressive symptoms.^10^, ^17^ In the present study, depressive symptoms were measured using the BDI‐II^27^, ^28^ to examine construct validity. Items are evaluated on a four‐point Likert scale from 0 to 3, based on symptom severity over the last two weeks. Total scores range from 0 to 63. The severity of depressive symptoms is determined from the total score. Higher scores indicate more severe depressive symptoms. We used the Japanese version of the BDI‐II,^29^ which has been confirmed to have high validity and reliability using back translation. (Cronbach's alpha for the original Japanese version of the scale is 0.87) In the present study, the BDI‐II showed high internal consistency (Cronbach's α = 0.92).

#### 2.3.5 Objective assessment of self‐stigma by attending physician

To examine the association between scale scores and an external criterion, we asked the attending physician to objectively assess self‐stigma levels in PWE using a 10‐point scale. The assessment comprised the physician's response to the following question: “How would you rate your patient's level of self‐stigma?” Responses were on a scale of 1 to 10, from “The patient has no self‐stigma at all” (1) to “The patient has very strong self‐stigma” (10). Higher scores indicate greater self‐stigma.

### 2.4 Ethical considerations

The study was conducted following the approval of the study protocol by the Institutional Review Board of Saitama Medical Center, Saitama Medical University (approval number: 2374). Participation was voluntary, and information was collected anonymously after obtaining written consent from each respondent. Participants were assured that their data would be kept confidential throughout the data collection period.

### 2.5 Data analysis

Statistical analyses were performed using SPSS version 24 (IBM Corp). Descriptive data were expressed in means, standard deviations, ranges, and percentages. The internal consistency of the ESSS was calculated using Cronbach's α, and the test‐retest reliability was evaluated using the intraclass correlation coefficient. Exploratory factor analysis (EFA) was conducted to examine the factor structure of the ESSS. To examine the scale's construct validity, Pearson's correlation coefficients were calculated to examine the association between ESSS scores and self‐esteem, depressive symptoms, and objective physician evaluation. To examine the association between ESSS and demographic variables (age, sex, education, employment and marital status, and residential status), Pearson's correlation coefficient was calculated or a *t* test conducted. Significance probability was set at *P* < .05.

## 3 RESULTS

### 3.1 Participant characteristics

Questionnaires were returned from 102 participants (response rate: 51%). After excluding two participants with incomplete questionnaires, data for 100 participants were included in the final analysis (53 women, 47 men; mean age: 39.86 years; standard deviation: 17.45 years). Objective physician assessments were obtained for 54 participants. Other patient characteristics and scale scores are shown in Table 1.

**TABLE 1:** Participant characteristics and scale scores

Variables	Mean (SD)
Age, years	39.86 (17.45)
Range	18‐84
Sex	
Female	53 (%)
Male	47 (%)
Length of education	13.81 (2.41)
Employment status	
Employed	45 (%)
Unemployed	48 (%)
On leave	7 (%)
Marital status	
Single	64 (%)
Married	36 (%)
Living style	
Alone	25 (%)
With other	75 (%)
ESSS	19.44 (5.88)
Internalization of stigma	8.75 (3.25)
Societal incomprehension	6.07 (1.63)
Confidentiality	4.62 (2.11)
RSES	29.11 (9.78)
BDI‐Ⅱ	12.97 (11.17)
Obj.	4.09 (2.38)

Abbreviations: BDI‐II, Beck Depression Inventory‐Second Edition; ESSS, Epilepsy Self‐Stigma Scale; Obj., Objective assessment of self‐stigma by attending physician; RSES, Rosenberg Self‐Esteem Scale; SD, standard deviation.

### 3.2 Exploratory factor analysis

We performed EFA to establish the number of dimensions of the 18‐item ESSS prototype scale, in line with recent recommendations.^30^ The maximum likelihood estimation factor extraction method was used. The value of the Kaiser‐Meyer‐Olkin test was 0.90, and Bartlett's test of sphericity was significant (*P* < .001).

These findings indicated that the correlations between the variables were high enough to provide a reasonable basis for EFA. Three factors were retained according to the Kaiser‐Guttman criterion (eigenvalues <1) and the scree test (number of factors before an abrupt drop in eigenvalues). The eigenvalues of the three factors were 8.09, 1.35, and 1.20. After deleting four items (3, 9, 14, and 13) with low factor loadings (<0.4), we repeated the EFA using a Promax rotation, excluding items with loadings across multiple factors, until an interpretable structure was obtained. Finally, eight items loading on three factors were extracted (Table 2).

**TABLE 2:** Exploratory factor analysis results (n = 100)

	Items (α = 0.87)	1	2	3			
Factor 1 Internalization of stigma (α = 0.81)							
7	When I hear news about traffic accidents related to epileptic seizures, I feel like I'm being told about myself.	**0.95**	−0.12	−0.13			
16	I feel discriminated against by others because of epilepsy.	**0.61**	0.00	0.15			
6	I feel myself different from others because of I have epilepsy.	**0.52**	0.25	0.03			
1	I feel sometimes embarrassed for epilepsy.	**0.48**	0.03	0.29			
Factor 2 Societal incomprehension (α = 0.76)							
5	Few people have the correct information about the disease of epilepsy.	−0.14	**0.91**	−0.05			
4	Ordinary people do not understand my suffering from epilepsy and the worry of seizures.	0.21	**0.65**	0.05			
Factor 3 Confidentiality (α = 0.85)							
12	I want to hide the fact that I go to hospital to receive therapy for epilepsy.	−0.08	−0.03	**0.99**			
8	It is hard to tell others that I have epilepsy.	0.05	−0.01	**0.77**			
	Interfactor correlation	1	2	3			
	1		0.66	0.67			
	2			0.45			
	3						
Excluded items							
2	I think many people have a bad image of epilepsy.						
3	I think my epileptic seizures are bothering others.						
9	I'm afraid of epileptic seizures.						
10	I have epilepsy so I can't do what I want to do (eg, sports, work, marriage, etc).						
11	I think epilepsy causes people to worry more than necessary.						
13	Epilepsy is thought to be a special illness that cannot be cured by the ordinary people.						
14	I have a bad image about epilepsy.						
15	I think epilepsy is attributed to me by the ordinary people.						
17	I find it hard to keep taking medicine because of epilepsy.						
18	I can't live the way I want because of epilepsy.						

Note

Bold indicates each inter factors correlation.

Extraction method: maximum likelihood estimation; rotation method: Promax.

Abbreviation: ESSS, Epilepsy Self‐Stigma Scale.

Following author discussion, we interpreted and named these factors as follows. Factor 1 was named “internalization of stigma” and comprised four items (1, 6, 7, and 16). This factor represents the extent to which PWE have emotional experiences related to internalized stigma. Factor 2 was named “societal incomprehension” and comprised two items (4 and 5). This factor represents the extent to which PWE feel separated from their surroundings. Factor 3 was named “confidentiality” and comprised two items (8 and 12). This factor represents the extent to which PWE wish to hide the fact that they have epilepsy.

### 3.3 Internal consistency reliability

To examine the internal consistency reliability of the ESSS, we calculated Cronbach's alpha coefficient. The total eight‐item scale showed a high level of internal consistency (α = 0.87), and each of the three subscales showed sufficient internal consistency (α = 0.76‐0.85).

### 3.4 Test‐retest reliability

To examine the test‐retest reliability of the ESSS, we calculated the intraclass correlation coefficient for the 21 participants who completed the questionnaire twice (at an interval of approximately one to four weeks) during the study period. The correlations were 0.89 for the total ESSS score, 0.90 for the first factor (internalization of stigma), 0.72 for the second factor (societal incomprehension), and 0.90 for the third factor (confidentiality), indicating sufficient test‐retest reliability.

### 3.5 Construct validity

To examine the construct validity, Pearson's correlation coefficients for the association between the ESSS and RSES, BDI‐II, and objective physician assessment were calculated (Table 3). The results showed that there were significant moderate correlations between the total ESSS score and RSES and BDI‐II scores (r = −0.47, *P* < .001; r = 0.53, *P* < .001, respectively). There were significant moderate correlations between scores on the ESSS subscale internalization of stigma and RSES and BDI‐II scores (r = −0.52, *P* < .001; r = 0.55, *P* < .001, respectively). The societal incomprehension score showed a significant weak correlation with the RSES score (r = −0.30, *P* < .01) and a significant moderate correlation with the BDI‐II score (r = 0.43, *P* < .001). Confidentiality showed a significant weak correlation with RSES and BDI‐II scores (r = −0.28, *P* < .01; r = 0.30, *P* < .01, respectively). However, physician assessment correlated only with the confidentiality factor (a significant weak positive correlation; r = 0.32, *P* < .05).

**TABLE 3:** Correlations between ESSS scores and other measures (n = 100)

	RSES	BDI	Obj
ESSS	−0.47^***^	0.53^***^	0.10
Internalization of stigma	−0.52^***^	0.55^***^	0.03
Societal incomprehension	−0.30^**^	0.43^***^	0.00
Confidentiality	−0.28^**^	0.30^**^	0.32^*^

Note

^*^ *P* < .05, ^**^ *P* < .01, ^***^ *P* < .001.

Abbreviations: BDI‐II, Beck Depression Inventory‐Second Edition; ESSS, Epilepsy Self‐Stigma Scale; Obj., Objective assessment of self‐stigma by attending physician; RSES, Rosenberg Self‐Esteem Scale; SD, standard deviation.

### 3.6 Association between demographic variables and ESSS score

There was no significant correlation between total or subscale ESSS scores and age. However, ESSS total scores and confidentiality scores showed a significant weak positive correlation with the length of education (r = 0.28, *P* < .01; r = 0.36, *P* < .001, respectively). A longer educational history was associated with greater confidentiality self‐stigma. The *t* test results showed no significant differences in total ESSS scores and subscale scores according to sex, employment or marital status, or living style.

## 4 DISCUSSION

### 4.1 Reliability and validity of the ESSS

The aims of the present study were to develop an epilepsy self‐stigma scale (the ESSS) to measure the extent of self‐stigma in PWE and to examine the scale's reliability and validity. The ESSS was shown to be sufficiently reliable. High internal consistency and test‐retest reliability were obtained, indicating that the ESSS has adequate reliability. The results of the EFA and correlations with related scales indicated that the ESSS has adequate construct validity. Among the subscales of the ESSS, societal incomprehension was weakly correlated with self‐esteem, and confidentiality was weakly correlated with self‐esteem and depression. One possibility is that differences in the subscales of the ESSS may affect the degree of correlation. The strongest correlations with psychosocial variables such as self‐esteem and depression have been found for internalized stigma among self‐stigma.^10^ Societal incomprehension has the component of perception of stigma, and confidentiality has the component of actions taken to avoid stigma. Societal incomprehension and confidentiality are considered to have large perceptive or behavioral components. Therefore, the difference in the degree of correlation with psychosocial variables may have occurred. Although the degree of correlation was weak, the positive and negative relationships were consistent with those of previous studies, and there were no problems with validity.

The ESSS is an eight‐item scale with subscales comprising three factors: internalization of stigma (internalized stigma), societal incomprehension (perception of stigma), and confidentiality (actions taken to avoid stigma). The total scale can be used to assess self‐stigma in PWE. Higher scores indicate greater self‐stigma caused by epilepsy. Furthermore, the subscale scores indicate which aspects of self‐stigma are strongest in PWE.

### 4.2 Usefulness of questionnaires for clinical application

In general epilepsy treatment, it is difficult to objectively evaluate the level of self‐stigma of epilepsy patients using only a short examination or general medical consultation. We found that ESSS scores were not related to objective physician evaluation of self‐stigma. The ESSS may objectively clarify subjective internal stigma in PWE that is undetectable to physicians. As such, the ESSS could provide an easier way to assess epilepsy self‐stigma in clinical situations. Additionally, ESSS results could inform medical consultations and interventions for patients. In this study, we were unable to confirm the ESSS cutoff point for severe self‐stigma. Additional studies are needed to test the scale on patients with high self‐stigma. If physicians can identify patients with high self‐stigma using the ESSS, they could discuss this with patients or refer them for psychiatric or psychological consultations. We believe that the ESSS would be very useful in clinical settings.

High self‐stigma tends to be associated with depression and low self‐esteem; reducing self‐stigma can improve depressive symptoms and low self‐esteem in patients, and ultimately improve their QOL.^10^, ^17^ Because self‐stigma is associated with QOL, evaluation of self‐stigma and interventions to reduce it are important in the treatment of epilepsy.

Interestingly, studies in other countries have reported that younger age is associated with greater perception of stigma.^14^, ^16^, ^17^ In the present survey, there was no significant age difference in self‐stigma. However, stigma is a combination of social stigma and self‐stigma, so the ESSS findings may not necessarily match those from other self‐stigma measures.

Regarding educational history, findings from non‐Japanese studies indicate that a lower education level is associated with greater stigma owing to lack of knowledge. However, the present findings showed that a longer educational history was associated with a stronger tendency for self‐stigma. Although self‐stigma is internalized social stigma, it is possible that accurate information about epilepsy has not been widely disseminated in Japan; therefore, individuals with a longer educational history are more likely to experience (internalized) prejudice.

A study from the USA showed that high levels of courtesy stigma in families are associated with self‐stigma in PWE and with lower QOL.^19^ If a patient's self‐stigma is strengthened by courtesy stigma in the family, it makes it more difficult to obtain social resources.^31^ We hope that the ESSS will be used to identify stigma in both PWE and their families.

Self‐stigma is internalized stigma that can be reduced using cognitive therapeutic interventions. We plan to conduct a longitudinal study to determine if the ESSS is a useful effect index of interventions such as psychosocial education programs. We plan to report these findings in the future.

### 4.3 Limitations

The “gold standard” for scale validation is to confirm construct validity by comparing the measure with similar scales. However, there are currently few established Japanese versions of self‐stigma scales. Therefore, we were limited to examining construct validity using self‐esteem and depression measures. Some studies have shown that stigma perception is associated with the severity of epileptic seizures,^13^, ^15^, ^16^ but we did not assess seizure frequency in this study. We aim to use this questionnaire in a future study to investigate the relationship between seizures and self‐stigma.

Besides, as a result of the EFA, 10 items were removed. The major reason is the factor loadings spanned multiple factors. The ESSS scale items were developed based mainly on the results of qualitative analysis, rather than existing scales. It has been pointed out that the components of self‐stigma are complex and overlapping. Overlapping the subscales, many items wad excluded. However, it is significant that we could create a scale consisting of multiple components based on the narratives of PWE. In the future, it will be necessary to refine the scale items, referring to the theoretical framework of self‐stigma.

## 5 CONCLUSIONS

The present findings demonstrate that the Japanese version of the ESSS is reliable and valid and could therefore be used to reliably measure internalized stigma in Japanese PWE. A full list of items used in the final version of the ESSS is shown in Tables S2 (English) and Table S3 (Japanese).

We currently run a psychosocial education program for epilepsy patients and their supporters.^32^ A previous study showed that lower perceived stigma in parents of children with epilepsy was associated with more positive feelings, fewer worries, and more family leisure activities.^16^ Having accurate information about epilepsy is related to lower self‐stigma; the ESSS could be used as a self‐stigma evaluation tool in the development of psychosocial education programs. The scale could help researchers assess perception of self‐stigma in PWE and measure how these changes over time. The scale also permits an objective quantitative evaluation and so could be used to assess the effects of public health interventions aimed at reducing self‐stigma. We hope that this scale will help clinicians and researchers in Japan better understand the internalized stigma experienced by PWE.

## CONFLICT OF INTEREST

None of the authors has any conflict of interest to disclose. We confirm that we have read the Journal's position on issues involved in ethical publication and affirm that this report is consistent with those guidelines.

## ETHICAL STATEMENT

​Authorship of the paper: Authorship have been limited to those who have made a significant contribution to the conception, design, execution, or interpretation of the reported study. Originality and plagiarism: The authors have written entirely original works. Data access and retention: Authors prepared to provide public access to data. Multiple, redundant or concurrent publication: Authors have not in general publish manuscripts describing essentially the same research in more than one journal or primary publication. Fundamental errors in published works: When an author discovers a significant error or inaccuracy in her own published work, it is the author's obligation to promptly notify the journal editor or publisher and cooperate with the editor to retract or correct the paper.

## Supporting information

Table S1

Table S2

Table S3

## Data Availability

The study data are not publicly available.
