# Supplementary material for: Development and validation of the Epilepsy Self‐Stigma Scale
Source: Epilepsia Open. 2021 Oct 26;6(4):748–56. doi: 10.1002/epi4.12547 (PMC8633466; doi:10.1002/epi4.12547)
Supplement: Supplementary file 1 — Table S1 [file EPI4-6-748-s003.docx]

**TABLE S1.** Epilepsy Self-Stigma Scale prototype version (18 items)

| Item No. | Item |
| --- | --- |
| 1 | I feel sometimes embarrassed for epilepsy. |
| 2 | I think many people have a bad image of epilepsy. |
| 3 | I think my epileptic seizures are bothering others. |
| 4 | Ordinary people do not understand my suffering from epilepsy and the worry of seizures. |
| 5 | Few people have the correct information about the disease of epilepsy. |
| 6 | I feel myself different from others because I have epilepsy. |
| 7 | When I hear news about traffic accidents related to epileptic seizures, I feel like I'm being told about myself. |
| 8 | It is hard to tell others that I have epilepsy. |
| 9 | I'm afraid of epileptic seizures. |
| 10 | I have epilepsy so I can’t do what I want to do (e.g., sports, work, marriage). |
| 11 | I think epilepsy causes people to worry more than necessary. |
| 12 | I want to hide the fact that I go to hospital to receive therapy for epilepsy. |
| 13 | Epilepsy is thought to be a special illness that cannot be cured by ordinary people. |
| 14 | I have a bad image about epilepsy. |
| 15 | I think epilepsy is attributed to me by ordinary people. |
| 16 | I feel discriminated against by others because of epilepsy. |
| 17 | I find it hard to keep taking medicine because of epilepsy. |
| 18 | I can’t live the way I want because of epilepsy. |

**Note**

Items in bold were included in the final version of the scale.
