# Supplementary material for: Development and validation of the Epilepsy Self‐Stigma Scale
Source: Epilepsia Open. 2021 Oct 26;6(4):748–56. doi: 10.1002/epi4.12547 (PMC8633466; doi:10.1002/epi4.12547)
Supplement: Supplementary file 2 — Table S2 [file EPI4-6-748-s002.docx]

**TABLE S2.** Epilepsy Self-Stigma Scale (eight items)

This questionnaire will ask you about what you think about having epilepsy.

For each of the following questions, please choose the most appropriate number from the scale to the right of each sentence.

|  | **Strongly Disagree** | **Disagree** | **Agree** | **Strongly**  **Agree** |
| --- | --- | --- | --- | --- |
| 1. When I hear news about traffic accidents related to epileptic seizures, I feel like I’m being told about myself. | 1 | 2 | 3 | 4 |
| 2. I feel discriminated against by others because of epilepsy. | 1 | 2 | 3 | 4 |
| 3. I feel sometimes embarrassed for epilepsy. | 1 | 2 | 3 | 4 |
| 4. I feel myself different from others because I have epilepsy. | 1 | 2 | 3 | 4 |
| 5. Ordinary people do not understand my suffering from epilepsy and the worry of seizures. | 1 | 2 | 3 | 4 |
| 6. Few people have the correct information about the disease of epilepsy. | 1 | 2 | 3 | 4 |
| 7. It is hard to tell others that I have epilepsy. | 1 | 2 | 3 | 4 |
| 8. I want to hide the fact that I go to hospital to receive therapy for epilepsy. | 1 | 2 | 3 | 4 |
