# Supplementary material for: Development and validation of the Epilepsy Self‐Stigma Scale
Source: Epilepsia Open. 2021 Oct 26;6(4):748–56. doi: 10.1002/epi4.12547 (PMC8633466; doi:10.1002/epi4.12547)
Supplement: Supplementary file 3 — Table S3 [file EPI4-6-748-s001.docx]

**TABLE S3.** Epilepsy Self-Stigma Scale (eight items) Japanese version

**てんかんセルフスティグマスケール**

　この質問票は，あなたの「てんかん」という病気に対して，あなた自身がどう思っているか，意見をお伺いします。下の4段階の数字を使って，以下の文章にどの程度そう思うか，あるいはそう思わないかを，文章の右にある数字のうち最も当てはまると思うものを1つ選んでください。

|  | 全くそう思わない | 少し  そう思う | そう思う | 非常に  そう思う |
| --- | --- | --- | --- | --- |
| １．てんかん発作に関連する交通事故などのニュースを聞くと，自分のことをいわれているように感じる | 1 | 2 | 3 | 4 |
| ２．てんかんのために，周囲から差別される | 1 | 2 | 3 | 4 |
| ３．てんかんがあると，恥ずかしい思いをすることがある | 1 | 2 | 3 | 4 |
| ４．てんかんがあることで，自分は普通の人とは違うと感じる | 1 | 2 | 3 | 4 |
| ５．てんかんの苦痛や発作への心配は他の人にはなかなか理解してもらえない | 1 | 2 | 3 | 4 |
| ６．てんかんという病気について，正しい情報を持っている人が少ない | 1 | 2 | 3 | 4 |
| ７．てんかんであることを，他の人には言いにくい | 1 | 2 | 3 | 4 |
| ８．てんかんで通院・治療していることは隠しておきたい | 1 | 2 | 3 | 4 |
